# Atypical Prolonged Clinical Course of Blackleg in Nelore Cattle

**DOI:** 10.3390/ani16162503

**Published:** 2026-08-11

**Authors:** Gabrielle Araújo Rodrigues dos Santos, Braian Rombaldo de Oliveira, Juliana Portela Gonçalves Fagundes, Gabriel Costa Silva, Kamille Jorge Estevam, Larissa Martarella de Souza Mello, Renan Contini de Freitas, Gabriella Lima Santos, Letícia Iorio Lamim, Claudia Del Fava, Simone Miyashiro, Daniela Becker Birgel, Eduardo Harry Birgel Junior

**Affiliations:** 1 Department of Veterinary Medicine, School of Animal Science and Food Engineering, University of São Paulo, Pirassununga 13635-900, SP, Brazil; gabia915@gmail.com (G.A.R.d.S.); dabirgel@usp.br (D.B.B.); 2 Department of Animal Reproduction, School of Veterinary Medicine and Animal Science, University of São Paulo (USP), Pirassununga 13635-900, SP, Brazil; 3 Department of Surgery, School of Veterinary Medicine and Animal Science, University of São Paulo (FMVZ-USP), São Paulo 05508-270, SP, Brazil; 4 Luiz de Queiroz College of Agriculture (ESALQ), University of São Paulo, Piracicaba, São Paulo 13418-900, SP, Brazil; 5 Biological Institute, São Paulo 04014-002, SP, Brazil; 6 Biological Institute—Animal Health Research and Development Division, São Paulo 04014-002, SP, Brazil; claudia.fava@sp.gov.br (C.D.F.); simone.miyashiro@sp.gov.br (S.M.)

**Keywords:** *Clostridium chauvoei*, lameness, myocarditis, myocyte necrosis, *Bos indicus*

## Abstract

Blackleg, caused by *Clostridium chauvoei*, is a severe infectious disease of cattle typically characterized by an acute or peracute course with rapid progression to death within 48 h after the onset of clinical signs. However, well-described reports of chronic or prolonged clinical evolution are rare. This study aimed to describe an outbreak with an atypical, prolonged course and to report the therapeutic management of affected animals. In March 2023, an outbreak occurred in an unvaccinated Nelore herd, with seven cattle showing clinical signs and one dying before veterinary intervention. Due to disease severity, four animals were referred to the Veterinary Hospital (HOVET) of the School of Veterinary Medicine and Animal Science, University of São Paulo (FZEA-USP), while others were managed on-farm. Clinical management included fluid therapy, systemic antimicrobial treatment, surgical debridement of necrotic tissues, drainage, and intensive wound care. Despite treatment, three animals died during hospitalization. One severely affected animal underwent partial amputation of a hind limb and survived, and it was discharged after 116 days of treatment. Diagnosis was confirmed by PCR, clinical examination, and necropsy findings. Overall, the clinical course observed differed from the typical acute presentation, with two animals surviving, suggesting that intensive medical and surgical management may improve outcomes in selected cases. Cardiac lesions were identified in necropsied animals, with exclusive involvement of the left ventricle, a finding that may warrant further investigation. This report highlights atypical manifestations of blackleg and contributes to the limited literature on prolonged cases.

## Simple Summary

Blackleg, caused by *Clostridium chauvoei*, is a severe infectious disease of cattle, typically characterized by an acute or peracute course leading to death within 48 h. Reports describing chronic progression are rare. This study reports an outbreak with an unusually prolonged clinical course and the therapeutic management of affected animals. In March 2023, an outbreak of blackleg occurred in an unvaccinated Nelore herd. Seven cattle developed clinical signs, and two died before or shortly after veterinary intervention. Four severely affected animals were referred to the Veterinary Hospital (HOVET) of the School of Veterinary Medicine and Animal Science, University of São Paulo (FZEA-USP). Despite intensive treatment, including fluid therapy, antimicrobial therapy, surgical debridement, drainage, and wound management, three animals died. Diagnosis was confirmed by PCR, clinical evaluation, and necropsy findings. The prolonged clinical course observed contrasts with the classical acute presentation of blackleg. Two animals survived, demonstrating that intensive medical and surgical management may improve outcomes in selected cases. Cardiac lesions were identified in two of three necropsied animals, exclusively affecting the left ventricle. This report contributes to the limited literature on chronic blackleg and highlights the need for further studies on atypical clinical presentations and treatment strategies.

## 1. Introduction

Blackleg, caused by *Clostridium chauvoei* (*C. chauvoei*), is a serious infectious disease that primarily affects young cattle and, less commonly, sheep, goats, and wild animals [1,2,3,4,5], often leading to death within 12 to 48 h, as it is a disease with an acute or hyperacute course [4].

The main route of infection occurs through the phagocytosis of spores present in the environment, which are subsequently transported by macrophages from the intestine to the musculature and other tissues, showing a greater affinity for skeletal and cardiac muscle, where they remain in a latent state. This latency is maintained due to the anti-inflammatory profile exhibited by spore-infected cells. Conditions such as muscle trauma, in which tissue oxygen concentration decreases and lactic acid concentration increases [6,7], favor spore germination, bacterial multiplication, and the subsequent production of α-hemolysin, necrotoxin, β-deoxyribonuclease, γ-hyaluronidase, and δ-hemolysin. These toxins cause capillary damage and skeletal myocyte and myocardial necrosis [8,9].

The soluble antigens of *C. chauvoei* play crucial roles in the pathogenesis of blackleg, acting in a coordinated manner to cause extensive tissue damage. Alpha toxin possesses hemolytic and necrotizing properties, playing a key role in the destruction of red blood cells and the induction of muscle necrosis. Beta toxin degrades nuclear DNA, contributing to myocyte necrosis and cell death. Hyaluronidase (gamma toxin) degrades hyaluronic acid in connective tissue, facilitating the spread of the pathogen and toxins throughout the body. The delta toxin, a cytolysin, and toxin A form pores in the cell membrane, leading to the lysis of host cells. Finally, neuraminidase cleaves sialic acids from glycoproteins and glycolipids, promoting bacterial dissemination and tissue destruction by breaking down cellular barriers and releasing nutrients for the pathogen [5,10].

Clinical signs such as fever, anorexia, depression, lameness, and subcutaneous crepitus appear suddenly; in classic cases, edema and crepitus are present in muscle groups such as the pelvic limbs, diaphragm, and heart [3]. Although the typical clinical course is described as rapidly progressing and highly lethal, with acute or hyperacute progression, there are rare case reports of prolonged disease progression [2,8].

The diagnosis is based on clinical signs and postmortem findings and is confirmed by laboratory tests—such as culture and isolation of the pathogen, immunofluorescence, and immunohistochemistry—or molecular biology techniques, such as PCR [11].

The objective of this study is to discuss the clinical and therapeutic characteristics of an outbreak of blackleg that presented atypically, with a prolonged course of the disease—a feature rarely described in the literature. Thus, we aim to contribute to a better understanding of the different manifestations of the disease.

## 2. Case Report

**Figure 1:**
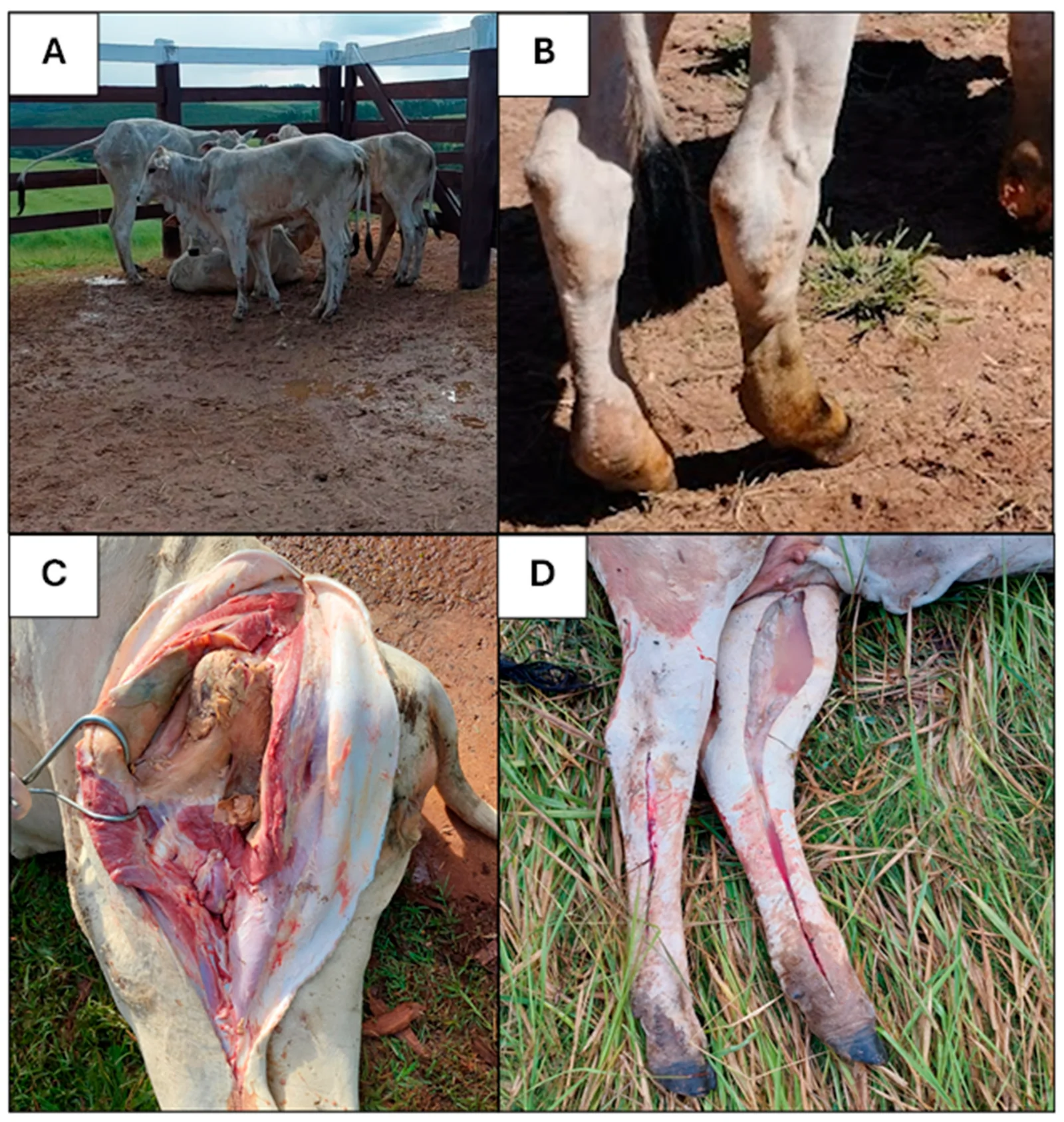
(**A**) Animals on the farm; (**B**) hind limb edema; (**C**) muscle necrosis; (**D**) postmortem edematous hind limbs.

In March 2023, a beef cattle farm requested veterinary assistance after purchasing 100 Nelore heifers aged between 12 and 24 months and weighing between 200 and 250 kg. The producer reported that the animals had been transported over a distance of 300 km and had no vaccination history against blackleg. One day after arrival, a heifer developed lameness and swelling of the hind limb musculature, subsequently progressing to death. In addition to edema and crepitus of the muscle masses, phlegmon was observed in the distal portion of the hind limbs (Figure 1).

**Figure 2:**
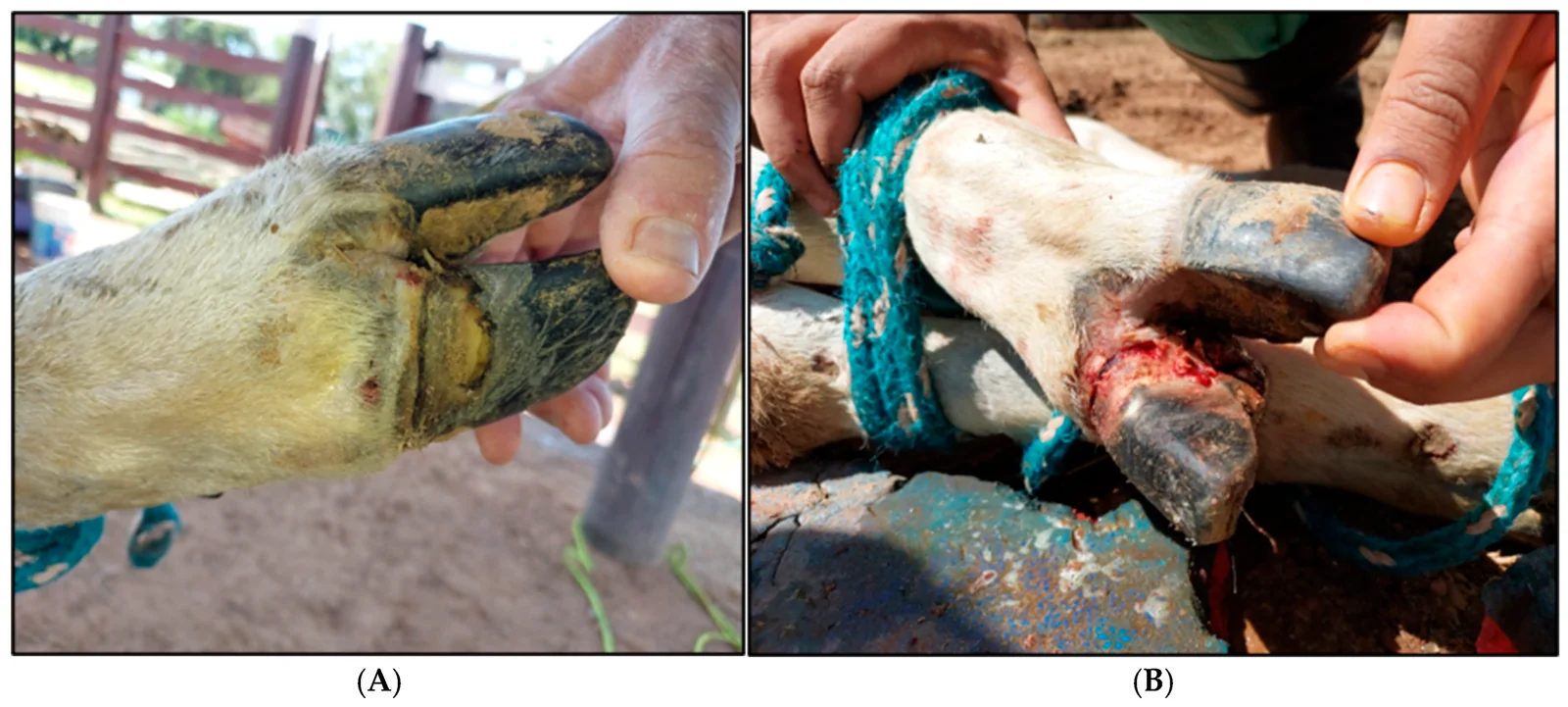
Hoof lesions observed during the clinical course of the disease. (**A**) Medial digit of the left hind limb showing severe edema and phlegmon of the coronary band. (**B**) Medial digit of the left hind limb showing an ulcerative lesion involving the coronary band, accompanied by partial loss of the hoof horn capsule.

In addition, six other animals exhibited similar clinical signs consistent with blackleg and were treated for seven days with penicillin (potassium benzylpenicillin 7500 IU/kg, procaine benzylpenicillin 7500 IU/kg, and benzathine benzylpenicillin 15,000 IU/kg, IM) combined with streptomycin (dihydrostreptomycin 6.25 mg/kg and streptomycin 6.25 mg/kg, IM). Furthermore, the entire herd received two doses of a vaccine containing inactivated clostridial cultures administered 30 days apart. The owner’s initial decision was to maintain treatment exclusively on the farm, with periodic visits performed to monitor the progression of the cases. During the clinical course of the disease, the affected animals developed hoof lesions characterized by severe edema, phlegmon, ulcerative lesions involving the coronary band, and partial loss of the hoof horn capsule (Figure 2).

The diagnosis of blackleg was based on the clinical signs of gas gangrene and was confirmed by PCR. The assay was performed at the Instituto Biológico of São Paulo. The DNA of *C. chauvoei* was detected in subcutaneous fluid and muscle tissue samples collected from two cattle, confirming the diagnosis of blackleg. Genetic material amplification was carried out using a multiplex PCR assay adapted from a previously described protocol [12], enabling the simultaneous detection of *C. chauvoei*, *C. haemolyticum*, *C. novyi* type A, *C. novyi* type B, and *C. septicum* based on the flagellin gene.

After 26 days from the onset of the outbreak, due to logistical difficulties such as the lack of adequate animal restraint facilities, limited resources for more invasive therapies, and the death of an additional animal on the farm, the four heifers with the most severe clinical conditions (Animals A, B, C, and D), among the five affected animals, were referred to the Veterinary Hospital (HOVET) of the School of Veterinary Medicine and Animal Science, University of São Paulo (FZEA-USP). The fifth animal (Animal E), which had a better prognosis because it presented milder clinical signs, remained on the farm. Physical and laboratory examinations were performed, including a complete blood count and serum biochemical profile, the results of which are presented in Table 1.

Animal A arrived at the hospital in critical condition and remained in permanent recumbency. It was treated with fluid therapy (10 L of Lactated Ringer’s solution, 5 g of Acetyl D-L-methionine, 2 g of choline chloride, and 30 g de glucose, daily wound care of the affected areas, and oxytetracycline hydrochloride at 20 mg/kg SID. Due to the severity of the lesions, Animals B and C underwent surgical treatment consisting of extensive muscle incision, wound lavage with a 3% aqueous hydrogen peroxide solution, debridement of necrotic tissue, immersion of the affected limbs in antiseptic baths containing a 0.01% potassium permanganate solution (1 g of potassium permanganate per 10 L of solution), and application of 2% iodine tincture once daily, in addition to oxytetracycline hydrochloride at 20 mg/kg SID. Animal D underwent amputation of the lateral claw of the left hind limb because of the development of distal necrosis and gangrene involving the distal interphalangeal joint. Owing to the extensive tissue destruction and irreversible joint involvement, digit amputation was considered the only therapeutic option. The procedure was performed using the classical Hannover open digit amputation technique [14], allowing healing by second intention. Briefly, a Gigli wire saw was used to transect the digit immediately distal to the coronary band. The distal and middle phalanges were removed together with all devitalized soft tissues, including the distal sesamoid bone and the deep digital flexor tendon. The articular cartilage covering the distal end of the proximal phalanx was removed, and the superficial and deep digital flexor tendons were transected as proximally as possible. Meticulous debridement of all necrotic and contaminated tissues was performed before the surgical wound was left open to allow drainage and healing by second intention. Postoperative management consisted of regular wound cleaning and bandage changes until complete granulation tissue formation and epithelialization were achieved.

**Figure 3:**
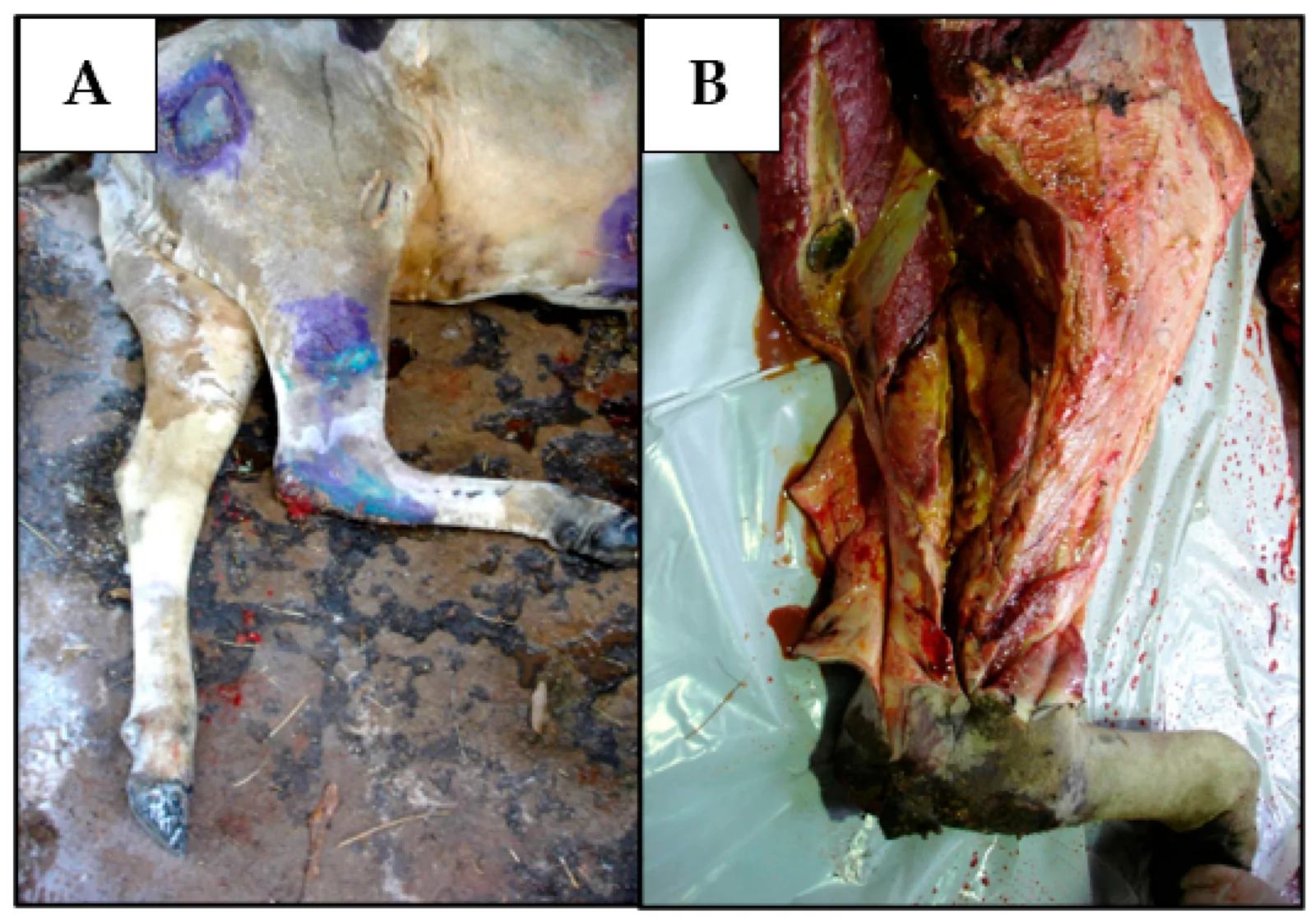
(**A**) Animal showing a “rabbit-leg” posture, with several areas of injury observed; (**B**) limb of the animal postmortem showing areas of necrosis and muscle rupture.

After 3 and 4 days of hospitalization (29 and 30 days after disease onset), Animals A and B died, respectively. Animal C was euthanized after 22 days of hospitalization (48 days after disease onset) due to the development of cardiac arrhythmia detected during cardiac auscultation, without further electrocardiographic characterization and progression of gastrocnemius muscle necrosis to complete rupture, resulting in a “rabbit-leg posture”. This posture is characterized by extension of the affected limb toward the ground, the inability to maintain the calcaneus in an elevated position, and contact of the plantar aspect of the tarsus with the ground, resulting in an approximately 90° angle between the tibia and the metatarsus (Figure 3).

The three hospitalized animals that died underwent routine postmortem examination at HOVET. During the necropsy of Animal C, samples of skeletal muscle, heart, lung, liver, kidney, spleen, and small intestine were collected for histopathological examination. Tissue fragments fixed in 10% buffered formalin were processed using standard histological techniques in an automatic tissue processor, including dehydration in graded ethanol, clearing in xylene, and paraffin embedding [15]. Subsequently, the tissues were embedded in paraffin blocks using histological cassettes. Sections of 3 μm thick were obtained using a rotary microtome, floated in a 53 °C water bath, and mounted on albumin-coated glass slides. The slides were then incubated in a ventilated oven at 63 °C until deparaffinization, stained with hematoxylin and eosin (H&E), and finally sealed with coverslips and synthetic resin.

**Figure 4:**
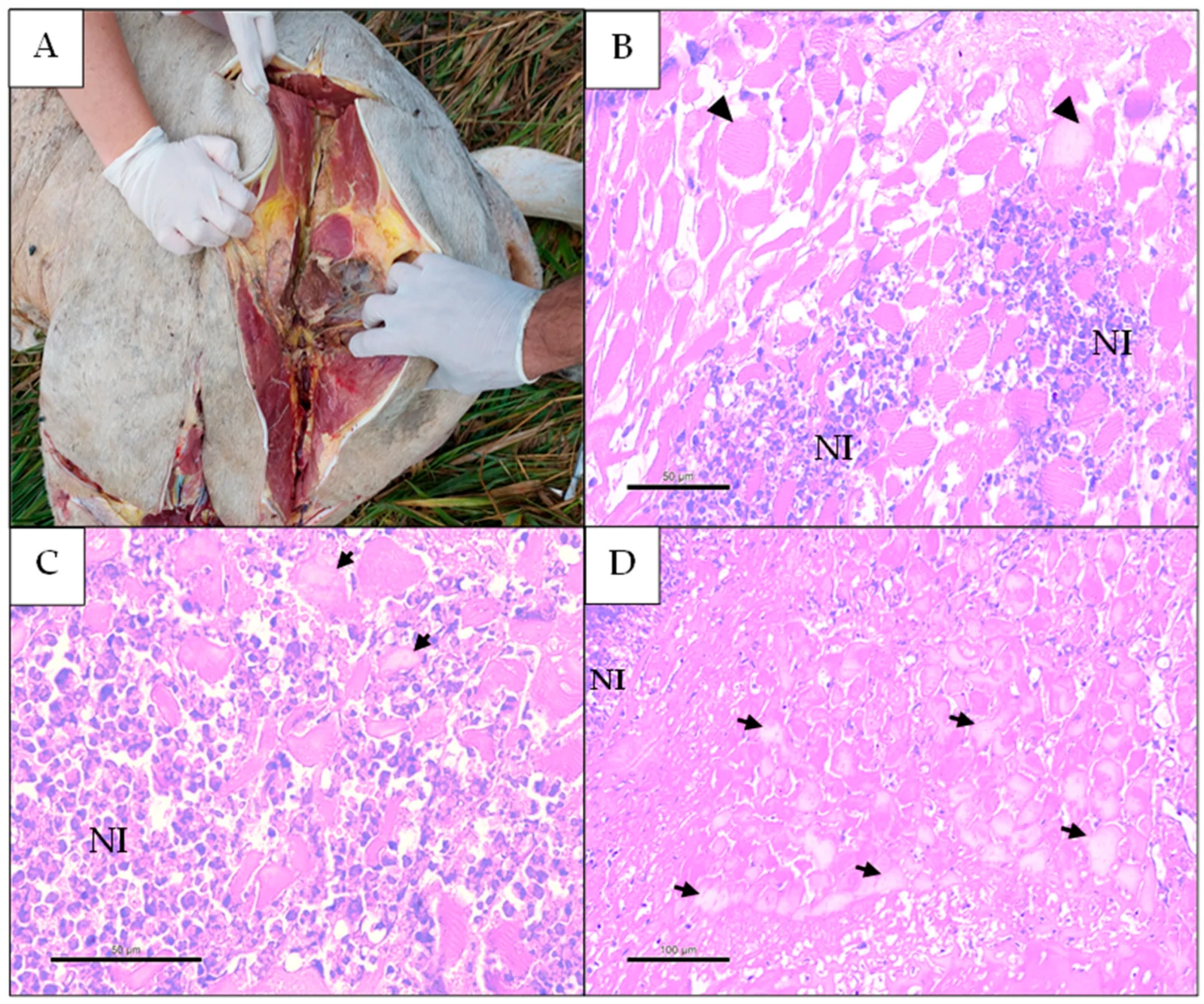
(**A**) Necrosis of the biceps femoris muscle. (**B**) Photomicrograph of a skeletal muscle section stained with hematoxylin and eosin (H&E) (630×). Myofibers undergoing coagulative necrosis with loss of striations (black triangles), accompanied by an intense and diffuse neutrophilic infiltrate (NI). (**C**) Photomicrograph of a skeletal muscle section stained with H&E (630×). Myofibers undergoing coagulative necrosis with loss of striations (black arrows) and an intense, diffuse neutrophilic infiltrate (NI) are observed. (**D**) Photomicrograph of a skeletal muscle section stained with H&E (200×). Extensive coagulative necrosis of myofibers (black arrows) accompanied by a neutrophilic infiltrate (NI).

Muscular necrosis was observed macroscopically in all animals. Histopathological examinations of the skeletal muscle revealed extensive areas of coagulative myofibrillar necrosis, with some retaining striations while others did not, accompanied by intense and diffuse neutrophilic infiltration (Figure 4).

**Figure 5:**
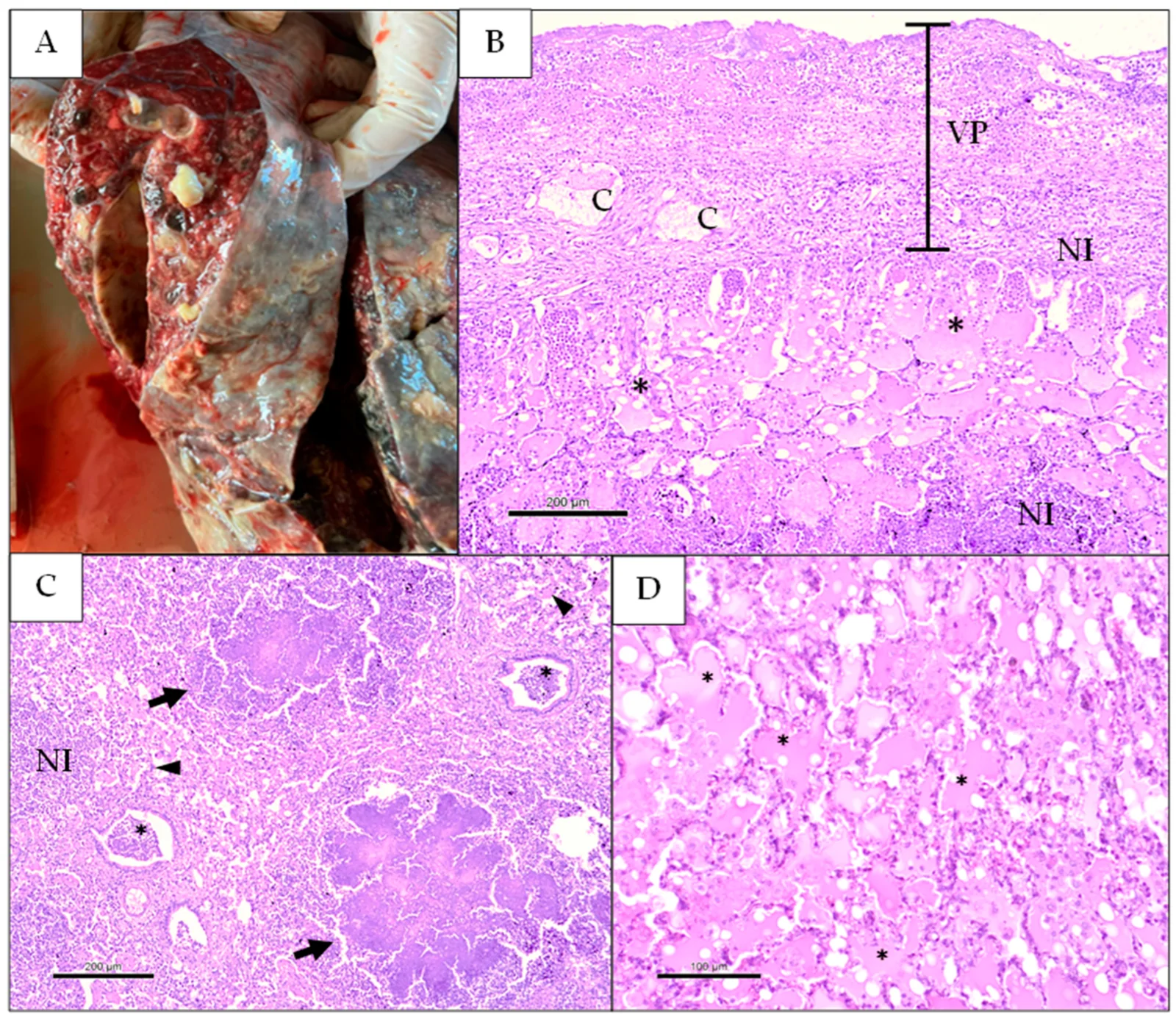
(**A**) Multiple abscesses in the left lung containing purulent material. (**B**) Photomicrograph of a lung section stained with hematoxylin and eosin (H&E) (100×). The visceral pleura (VP) is thickened and congested (**C**), and it exhibits an intense neutrophilic infiltrate (NI). The adjacent pulmonary parenchyma shows marked edema within the alveolar spaces (asterisks) and an intense, diffuse neutrophilic infiltrate (NI). (**C**) Photomicrograph of a lung section stained with H&E (100×). Two abscesses are observed within the pulmonary parenchyma (black arrows), along with desquamated epithelial cells and neutrophils (asterisks) in the bronchiolar lumen, thickening of the alveolar septa (black triangles), and an intense, diffuse neutrophilic infiltrate within the pulmonary parenchyma (NI). (**D**) Photomicrograph of a lung section stained with H&E (200×). Marked edema is observed within the alveolar spaces (asterisks).

Pulmonary lesions were identified in 100% (3/3) of the heifers and included congestion, hemorrhagic areas within the parenchyma, emphysema, and microabscesses. In the pleura, areas of intense neutrophilic infiltration and reactive mesothelial cells were observed. The pulmonary parenchyma exhibited moderate diffuse edema, multiple abscesses, severe congestion, abundant neutrophils, and foamy macrophages. Bronchioles contained desquamated epithelial cells and neutrophils within the lumen, along with loss of the epithelial brush border. However, because no lung samples were collected for bacteriological culture or PCR analysis, the etiology of these lesions could not be determined (Figure 5).

**Figure 6:**
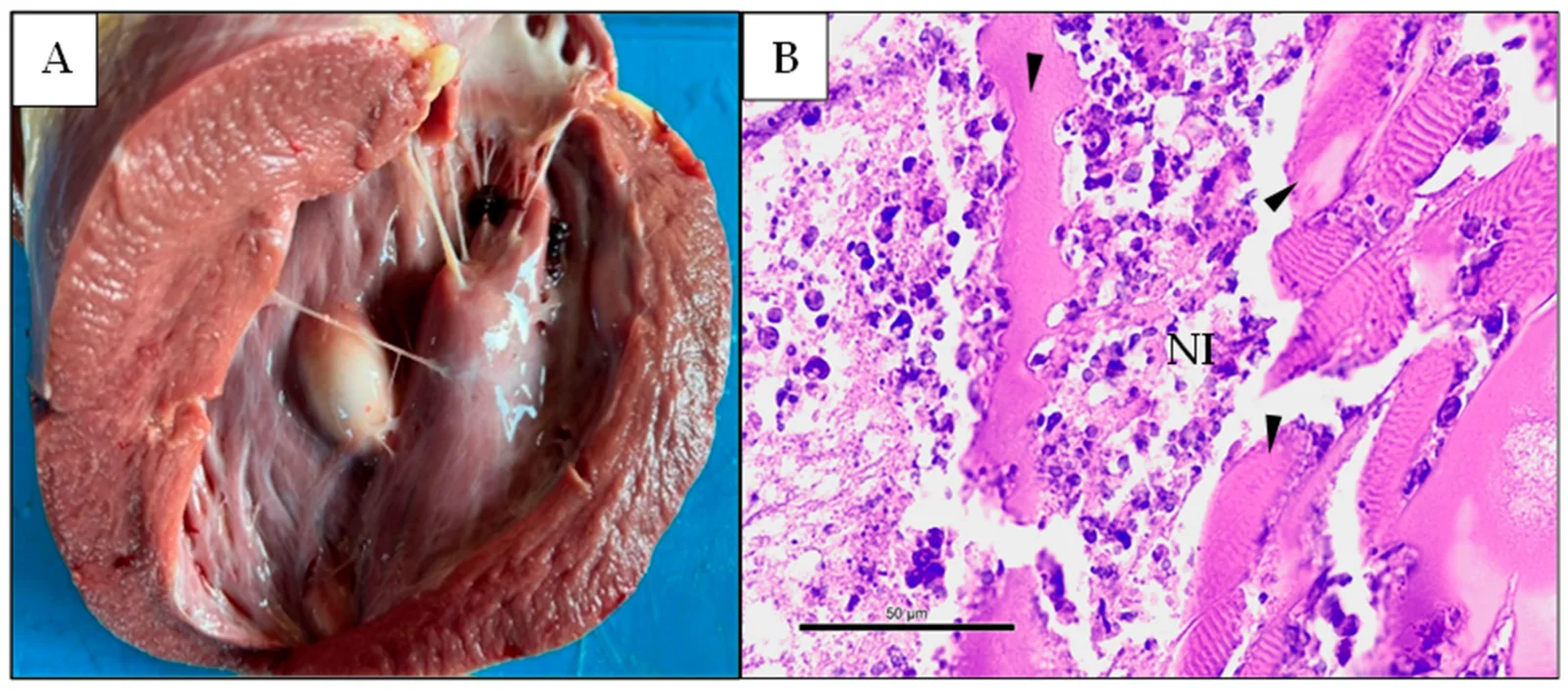
(**A**) Endocardial abscess. (**B**) Photomicrograph of a heart section stained with hematoxylin and eosin (H&E) (630×). Myocardial fibers undergoing coagulative necrosis with loss of striations (black triangles), accompanied by a neutrophilic infiltrate (NI).

Cardiac lesions were observed in 66.6% (2/3) of the animals and were characterized by myocardial pallor, abscesses on the ventricular endocardial surface and throughout the myocardium, and right ventricular dilation. Microscopically, extensive myocardial coagulative necrosis with fibrosis and neutrophilic infiltration was observed, with loss of striations in most affected fibers and preservation of striations in some areas (Figure 6).

**Figure 7:**
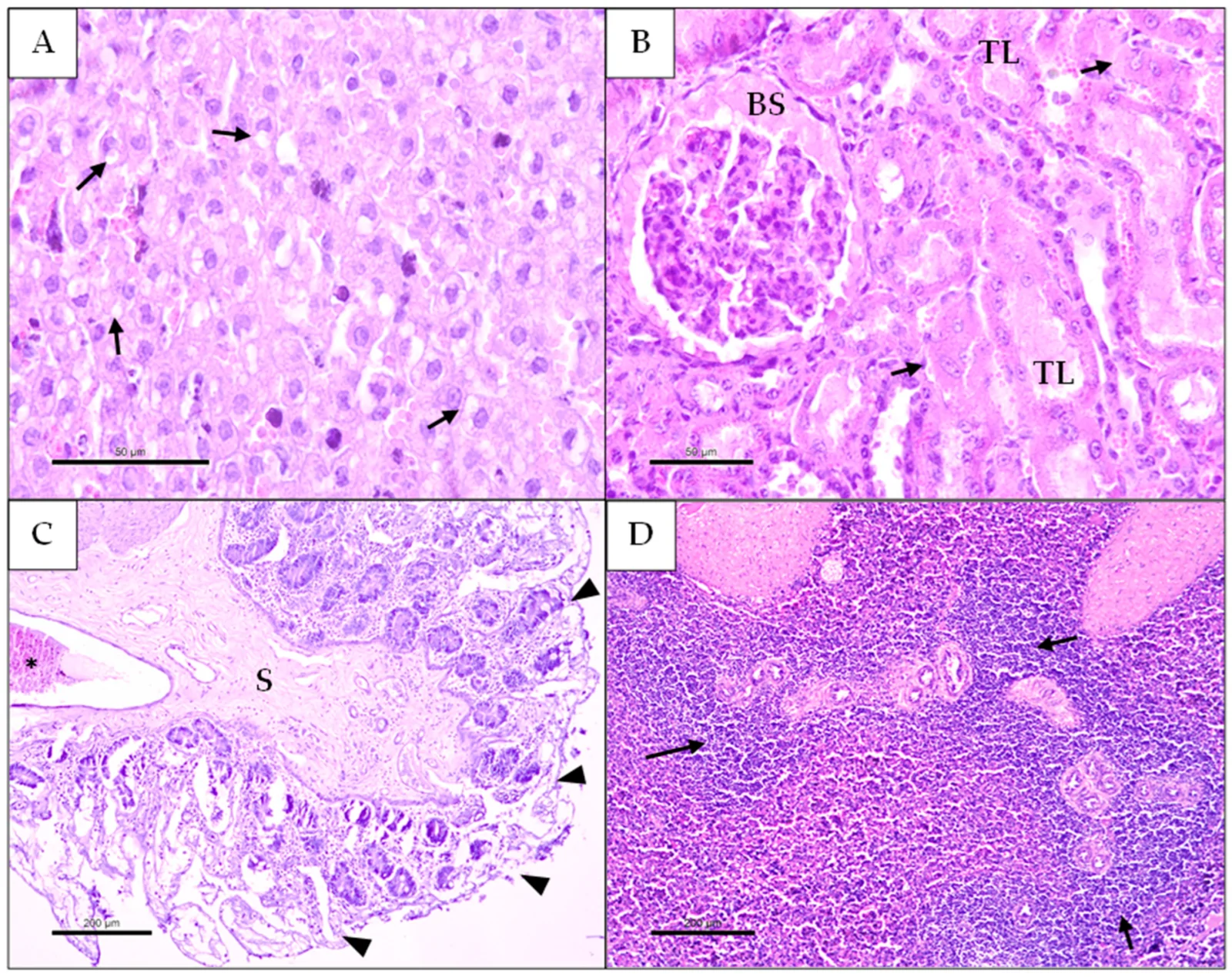
(**A**) Photomicrograph of a liver section stained with hematoxylin and eosin (H&E) (630×). Diffuse intracytoplasmic vacuolization of hepatocytes consistent with hydropic degeneration (black arrows) and hepatocellular swelling were observed. (**B**) Photomicrograph of a kidney section stained with H&E (630×). Coagulative necrosis of the renal tubules (black arrow) and eosinophilic material within the tubular lumen (TL) and Bowman’s space (BS), consistent with proteinuria, were observed. (**C**) Photomicrograph of a small intestine section stained with H&E (100×). Apical mucosal necrosis and adhesion of the intestinal villi (black triangles), submucosal edema (S), and congestion (asterisk) were observed. (**D**) Photomicrograph of a spleen section stained with H&E (100×). A moderate reaction of the splenic white pulp (black arrows) was observed.

In the liver, a severe and diffuse presence of hepatocytes containing intracytoplasmic vacuoles was observed, consistent with hydropic degeneration and hepatocellular swelling. The renal cortex exhibited extensive areas of coagulative necrosis within the convoluted tubules, as well as abundant eosinophilic material within the lumens of the convoluted tubules and glomerular spaces, consistent with proteinuria. In the spleen, moderate white pulp hyperplasia was observed. In the small intestine, the mucosa displayed apical necrosis and adhesion to intestinal villi, while the submucosa showed moderate congestion and edema (Figure 7).

**Figure 8:**
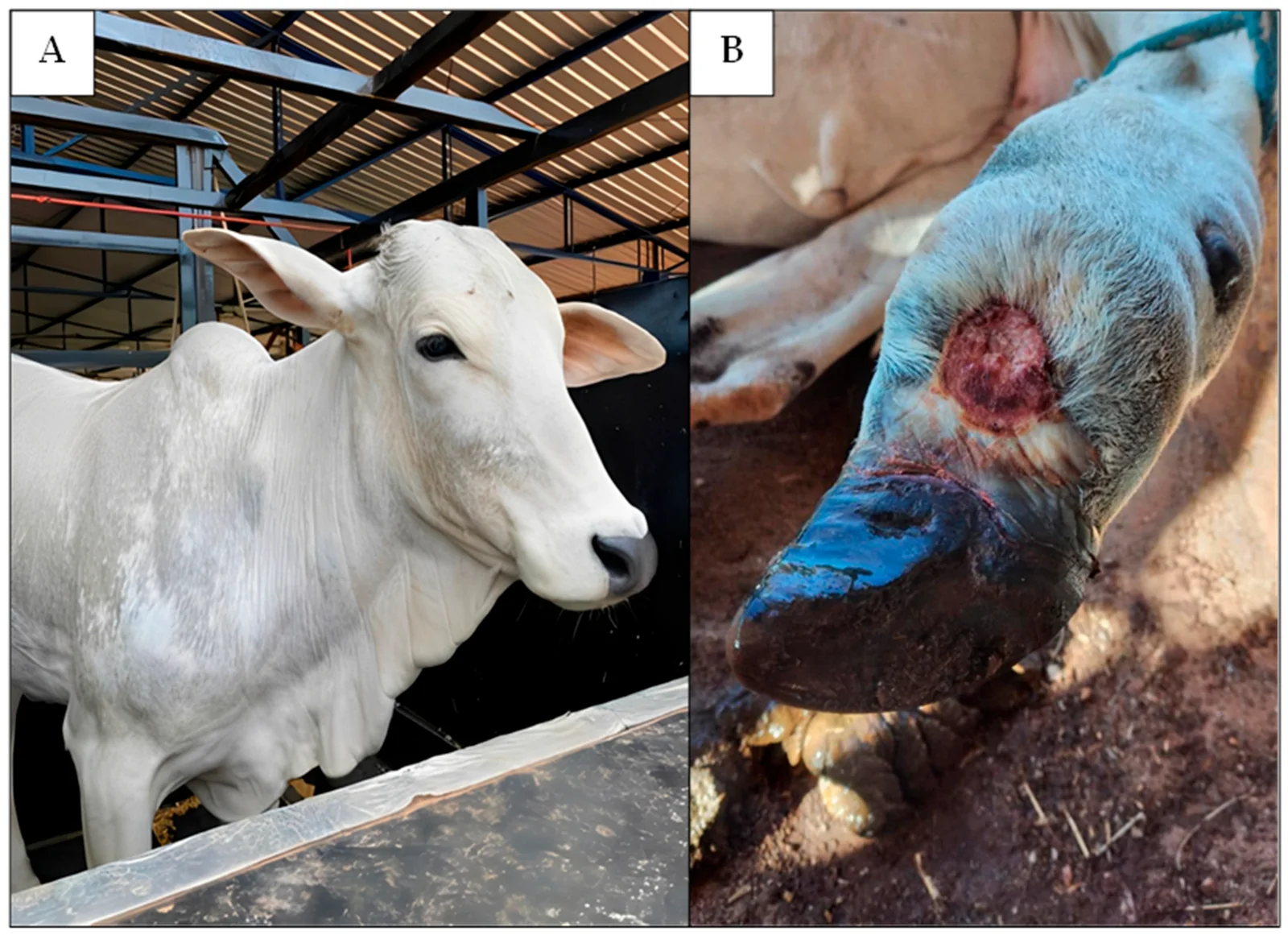
(**A**) Animal recovered after 90 days of hospitalization. (**B**) Affected limb in the final stage of healing following digit amputation and treatment.

Animal D showed satisfactory recovery following digit amputation and was discharged after 90 days of hospitalization/116 days after disease onset (Figure 8).

Animal E, which remained on the farm, demonstrated clinical improvement and returned to normal behavior.

The morbidity rate observed during this outbreak was 7% (7/100), whereas the mortality rate was 5% (5/100). The case-fatality rate was 71.4% (5/7), with 28.5% (2/7) of affected animals surviving. Additionally, 14.2% (1/7) of the animals developed clinical signs within 24 h after transportation, whereas 85.7% (6/7) developed signs between 24 and 48 h after arrival at the farm. Regarding muscle involvement, 28.5% (2/7) of the animals exhibited necrosis restricted to large muscle groups, such as the biceps femoris, gluteus medius, and semitendinosus muscles; 14.2% (1/7) showed lesions only distal to the hock, including the fetlock and hoof regions (distal limb phlegmon); 57.1% (4/7) exhibited necrosis in both anatomical regions.

## 3. Discussion

Although blackleg is a notifiable disease, as established by Normative Instruction No. 50 of September 24, 2013 [16], there are no recent official data from the Ministry of Agriculture, Livestock, and Supply (MAPA) on the occurrence of this disease in Brazil [17]. However, a study conducted by the Federal University of Santa Maria (UFSM) evaluated the etiological agents present in materials from animals infected with clostridia, sent to the laboratory between 1988 and 2007. The results indicated that *C. chauvoei* was identified in 28.45% (35/123) of the isolates of *Clostridium* spp., with the majority found in young cattle aged between 4 and 24 months [18]. These data indicate that, although underreported, blackleg remains a significant cause of mortality in young cattle, a finding consistent with the present report, in which affected animals were between 12 and 24 months old.

The classical literature describes blackleg as an acute or hyperacute disease, leading to death within 48 h of symptom onset [5,19]. In contrast, in this outbreak, we observed that only two of the seven affected animals (28.5%) died during this period; three animals (42.8%) died between 30 and 48 days after onset, and two of them (28.5%) recovered following the treatment administered. These results are consistent with previous reports [2,8], indicating that, although uncommon, the disease may follow a prolonged clinical course under specific conditions.

In Brazil, there is a scarcity of reports on the successful treatment of blackleg in cattle, highlighting the significance of the present case. Only two studies describing successful treatment outcomes were identified [2,8]. One reported the clinical cure of an animal after six months of disease progression through the use of penicillin therapy combined with extensive surgical incision of the affected musculature to promote tissue oxygenation, facilitate wound cleaning, and allow the application of dressings soaked in 2% iodine [8]. The other study similarly reported the complete recovery of a bovine after 32 days of disease progression; in this case, the diagnosis was based on clinical and epidemiological findings, and treatment consisted of penicillin-based antibiotic therapy combined with surgical incisions [2]. Considering that *Clostridium* spp. are obligate anaerobic microorganisms, the incision and drainage of the lesion, combined with the topical use of oxidizing agents such as hydrogen peroxide and potassium permanganate, have been reported as adjunctive approaches during the initial management of contaminated wounds [2,8]. These agents may contribute to reducing superficial bacterial load, promoting mechanical removal of organic debris, and increasing local oxygen availability transiently. These effects may help reduce the conditions favorable for spore germination and bacterial proliferation, particularly when combined with appropriate debridement and systemic antimicrobial therapy.

In the present report, penicillin-based antibiotic therapy was used during the first week of treatment, and from the moment the animals were admitted to the Veterinary Hospital, oxytetracycline was chosen as the antimicrobial agent. This choice was based on the drug’s availability, the total cost of treatment, and the fact that seven days of penicillin-based antibiotic therapy had already been administered on the farm. Drug treatment was combined with fluid therapy and surgical interventions, including wide incisions in the musculature and digit amputation, as well as wound management; these measures resulted in the prolonged survival of one animal for more than 40 days and the recovery of two animals. Although penicillins are traditionally the treatment of choice [4], the use of oxytetracycline has proven effective, shedding light on new therapeutic possibilities.

Of the three cases with prolonged disease progression that underwent necropsy, cardiac lesions were observed in 66.6% (2/3) of the animals. These findings are consistent with previous studies that have indicated a high prevalence of cardiac lesions associated with clostridial myositis in cattle, such as a study conducted in Tennessee, USA, which reported a prevalence of 70.3% [3], and a study conducted in California, USA, where cardiac lesions were found in 68.96% of the cases analyzed [19]. In the present report, cardiac lesions were observed exclusively in the left ventricle, involving both the endocardium and the myocardium. Regarding the location of the lesions, a higher incidence of pericarditis, epicarditis, or endocarditis on the right side of the heart and of myocarditis in the left ventricle has been reported [3]. Regarding the histopathological findings of the cardiac muscle, extensive multifocal areas of necrosis, fibrinoid degeneration, and polymorphonuclear leukocyte infiltration were observed, changes consistent with the literature [20,21,22].

In this report, isolated cardiac muscle involvement was not observed without skeletal muscle involvement. The occurrence of lesions solely in the cardiac muscle was previously described in five cases [20]. Although skeletal muscle necrosis is a hallmark feature of clostridial myositis, the cardiac changes described in this study highlight the potential for myocardial involvement to worsen clinical outcomes, including arrhythmias and heart failure.

In the literature, only two studies describe cases of animals that survived blackleg [2,8], both of which focused on therapeutic strategies and clinical recovery. However, no reports of necropsy findings in cases of prolonged disease course were found, making this the first report to document postmortem lesions in this type of disease presentation. Pulmonary lesions were also identified during the pathological examination. All heifers (3/3) presented pulmonary alterations, including congestion, hemorrhagic areas within the pulmonary parenchyma, emphysema, and microabscesses. Pulmonary lesions are not considered characteristic pathological findings of blackleg, although inflammatory pulmonary changes, including acute neutrophilic infiltration, interstitial pneumonia, edema, emphysema, and hemorrhage, have been reported in naturally infected animals [23]. However, because no lung samples were collected for bacteriological culture or PCR analysis, the etiology of these lesions could not be determined. Therefore, these findings cannot be conclusively attributed to *C. chauvoei* infection. Considering that the animals had not been vaccinated against pathogens associated with bovine respiratory disease (BRD), the pulmonary lesions may have been associated with concurrent respiratory infections caused by agents such as *Mannheimia haemolytica*, *Histophilus somni*, or *Mycoplasma* spp. Therefore, additional case reports and pathological investigations, including microbiological confirmation, are warranted to clarify whether pulmonary lesions may occasionally occur as part of the disease in cases of *C. chauvoei* infection or are more likely attributable to concurrent respiratory infections.

The leukogram findings, characterized predominantly by neutrophilia, were consistent with the gross and microscopic lesions observed in the skeletal muscles, myocardium, and lungs. Neutrophilia most likely resulted from increased bone marrow release of neutrophils in response to inflammatory mediators generated during tissue injury, followed by recruitment of these cells to sites of myocyte necrosis, where they participate in phagocytosis of necrotic debris and amplification of the acute inflammatory response through the release of cytokines and other inflammatory mediators [13]. The interpretation of hematological findings in cattle with blackleg is challenging because the disease progresses rapidly, and the hematological profile depends largely on the stage of infection at the time of blood sampling. Experimental studies have demonstrated a transient leukocytosis with neutrophilia during the early stages of infection, followed by leukopenia, neutropenia, and lymphopenia in terminal animals, together with an initial hemoconcentration and subsequent reductions in erythrocyte variables [24,25]. These alterations have been attributed to the cytotoxic and hemolytic effects of *C. chauvoei* exotoxins, combined with vascular injury associated with systemic toxemia [23]. The interpretation of these findings should also consider the kinetics of the bovine inflammatory response. Cattle possess a relatively small bone marrow reserve of mature neutrophils, and the development of a regenerative neutrophilic response generally requires approximately 3–5 days of increased granulopoiesis [26]. Because blackleg is typically a hyperacute disease, affected animals frequently die before an effective medullary response can develop. Therefore, the absence of marked leukocytosis or a regenerative left shift should not be interpreted as a mild inflammatory response but rather as a consequence of the rapid progression of the disease, which outpaces the kinetics of bovine granulopoiesis. Blood samples in the present study were collected upon hospital admission, approximately 30 days after the onset of clinical signs. Therefore, the hematological findings likely represent a more advanced stage of disease. The coexistence of animals with neutropenia and others with leukocytosis and neutrophilia may reflect differences in the stage of lesion evolution at the time of sampling. In addition, the extensive areas of myocyte necrosis may have predisposed affected tissues to secondary bacterial colonization, providing a persistent inflammatory stimulus capable of sustaining granulopoiesis and contributing to the development of leukocytosis and neutrophilia [26]. Although secondary bacterial infection was not investigated in the present study, this mechanism represents a plausible explanation for the hematological variability observed among the affected cattle.

An increase in total protein, fibrinogen, and globulin concentrations, accompanied by hypoalbuminemia, was also observed, consistent with a systemic inflammatory response. Fibrinogen and globulins are recognized as positive acute-phase proteins [27,28], whereas albumin is a negative acute-phase protein [29]. Collectively, these hematological and biochemical findings further support the presence of an ongoing inflammatory process associated with extensive muscular, myocardial, and pulmonary lesions. The microscopic hepatic lesions were consistent with the biochemical alterations indicative of hepatocellular injury and impaired hepatic function, including increased serum aspartate aminotransferase (AST) and gamma-glutamyl transferase (GGT) activity. Increased AST activity supported the presence of hepatocellular injury, whereas increased GGT activity suggested intrahepatic cholestasis secondary to inflammatory changes affecting the bile canaliculi [13]. Although necrotic lesions were observed in the kidneys, no biochemical evidence of renal dysfunction was detected. Increased serum creatine kinase (CK) activity correlated with the gross and microscopic findings of skeletal and cardiac muscle necrosis. This finding is consistent with previous reports, which identify CK as a sensitive biomarker of muscle injury due to its rapid release into the circulation following myocyte damage [13].

In this report, the affected animals exhibited severe lameness, but the amount of gas in the muscles was not sufficiently pronounced, resulting in clinical features that could be confused with other diseases, such as foot rot and interdigital phlegmon. This atypical presentation, combined with the fact that the deaths did not occur within 48 h, raised the hypothesis of endotoxemia or sepsis as possible causes of the losses. The detection of *C. chauvoei* by PCR, together with the clinical examination and necropsy findings, allowed these hypotheses to be ruled out and confirmed the diagnosis of blackleg.

During the diagnostic process, other clostridial myositis conditions should be considered, such as malignant edema and gas gangrene [30,31]. Other causes of sudden death, including anthrax, lead poisoning, toxic plant intoxication, and cerebral babesiosis, should be considered in differential diagnoses [5,30,31]. Furthermore, the presence of emphysematous edema associated with the characteristic muscular lesions of *C. chauvoei* further supported the diagnosis and helped exclude the principal differential diagnoses, which was subsequently confirmed by laboratory testing [5].

Although crepitant myocyte necrosis is not pathognomonic for blackleg, the epidemiological context and clinical presentation strongly supported *C. chauvoei* infection. Unlike malignant edema caused by *Clostridium septicum*, which is typically initiated by exogenous contamination of traumatized tissues, blackleg usually results from activation of latent *C. chauvoei* spores previously deposited in skeletal muscle following hematogenous dissemination [5]. Therefore, in the absence of an identifiable wound or other predisposing lesions, blackleg was considered the most likely clinical diagnosis before laboratory confirmation.

In the present report, no vaccination history was available for the affected animals. Although blackleg is commonly associated with a rapidly progressive and frequently fatal clinical course, not all animals in this outbreak progressed to death, and some cases exhibited a more chronic evolution. This prolonged clinical course may be associated with early and intensive antimicrobial therapy, initially with penicillin/streptomycin followed by oxytetracycline, which may have limited bacterial proliferation and subsequent toxin production.

In addition to therapeutic intervention, differences in the clinical evolution among animals may also be related to variations in the pathogenesis of *C. chauvoei* infection. The establishment of infection initially requires the ingestion of spores, followed by their hematogenous dissemination and subsequent germination in tissues subjected to trauma and local hypoxia, resulting in bacterial multiplication, toxin production, and myocyte necrosis [5]. Therefore, differences in the number and distribution of latent spores among tissues could influence disease severity. A lower spore burden may result in fewer sites of bacterial activation, leading to more localized lesions and a less severe clinical presentation, thereby contributing to the variability in clinical outcomes observed among affected animals.

Although passive immunity can be detected in calves for up to three months after ingestion of colostrum from cows vaccinated within 30 days before calving [5], the vaccination status of the dams may represent one possible factor influencing the observed outcomes. Vaccinated cows could have produced antibodies that were subsequently transferred to their calves through colostrum, potentially providing some degree of early protection. If such maternal immunity had occurred, previous exposure to colostral antibodies might have contributed to the subsequent immune response of these animals when later exposed to *C. chauvoei* spores as adults. In this context, previous immune stimulation may have influenced the host response to infection, potentially contributing, at least partially, to the control of bacterial proliferation and the variability in disease progression observed among animals.

Another possible, yet speculative, explanation could involve the concept of trained immunity [32]. In this form of immunity, innate immune cells acquire adaptive-like characteristics, resulting in long-term functional changes [32]. The development of trained innate immunity involves molecular mechanisms, metabolic reprogramming, and the activation of epigenetic pathways that allow certain functional modifications to persist over extended periods [32]. Although the existence and relevance of trained immunity in *C. chauvoei* infection remain unknown, it is conceivable that previous immune stimulation, including exposure to microbial components or other immunological stimuli during early life, could have influenced immune responsiveness later in life. Therefore, trained immunity may represent a potential contributing factor to the variability in disease severity observed in this outbreak. However, further experimental studies are required to determine whether such mechanisms play any role in protection against *C. chauvoei* infection or in modulating disease progression.

## 4. Conclusions

The present report demonstrates that blackleg caused by *Clostridium chauvoei* may present with a prolonged clinical course, differing from the classical hyperacute presentation commonly described in the literature. The survival of affected animals following intensive medical and surgical treatment suggests that recovery is possible in selected cases, particularly when therapeutic intervention is instituted early. Furthermore, the documentation of cardiac and pulmonary lesions associated with prolonged disease progression expands the current knowledge of blackleg pathogenesis, contributing to the improvement of future diagnostic and therapeutic approaches for this economically important disease. The use of PCR was essential for confirming the diagnosis, especially in animals presenting atypical clinical manifestations and delayed disease progression.

To the best of the authors’ knowledge, this is one of the first reports describing necropsy findings in cattle with prolonged blackleg progression, contributing to a better understanding of the pathological changes associated with this uncommon form of the disease. Additional studies are needed to clarify the factors influencing disease severity, survival, and treatment response, including the potential roles of host immunity and pathogen exposure load.

## Figures and Tables

**Table 1:** Hematological and biochemical results of blood samples collected upon the animals’ admission to the Veterinary Hospital.

Parameter	Animal A	Animal B	Animal C	Animal D	Reference [13]
Red blood cells (×10^12^/L)	9.46	5.66	6.89	8.32	5.0–10.0
Hemoglobin (g/dL)	10.0	6.6	6.50	8.50	8.0–15.0
Hematocrit (%)	31.6	21.7	21.0	29.0	24–46
Total Leukocytes (×10^3^/L)	13.50	21.20	7.60	11.60	4–12
Band Neutrophils (×10^3^/L)	0	0	0	0	0–0.12
Segmented Neutrophils (×10^3^/L)	9.04	12.29	2.43	6.49	0.6–4.0
Lymphocytes (×10^3^/L)	3.64	8.26	4.40	4.17	2.5–7.5
Monocytes (×10^3^/L)	0.81	0.63	0.76	0.93	0–0.09
Eosinophils (×10^3^/L)	0	0	0	0	0–2.4
Basophils (×10^3^/L)	0	0	0	0	0–0.2
AST (U/L)	144.4	263.2	N/D	88.4	60–125
GGT (U/L)	16.9	141.1	N/D	11.6	6.1–17.4
CK (U/L)	947.5	1267.9	N/D	617.2	0–350.0
Indirect Bilirubin (mg/dL)	0.64	1.00	N/D	0.10	0–1.40
Total Bilirubin (mg/dL)	0.84	1.20	N/D	0.20	0–1.60
Total Protein (g/dL)	9.80 ^1^	6.00 ^1^	10.40 ^1^	9.60 ^1^	6.7–7.5
Albumin (g/dL)	1.87	1.40	N/D	2.09	2.5–3.8
Globulins (g/dL)	7.93	4.60	N/D	7.51	3.0–3.5
Fibrinogen (mg/dL)	800 ^1^	400 ^1^	1200 ^1^	600 ^1^	100–600
Urea (mg/dL)	40.6	45.7	N/D	31.4	20–50
Creatinine (mg/dL)	1.05	1.15	N/D	1.56	0.5–2.2

N/D = not determined due to damage to the serum sample; ^1^ fibrinogen and total protein measurements were performed using plasma samples.

## Data Availability

The original contributions presented in this study are included in the article.

## Abbreviations

The following abbreviations are used in this manuscript:

FZEA-USP	Faculty of Animal Science and Food Engineering, University of São Paulo
PCR	Polymerase Chain Reaction
DNA	Deoxyribonucleic Acid
IM	Intramuscular
HOVET	Veterinary Hospital
MAPA	Ministry of Agriculture, Livestock, and Supply
UFSM	Federal University of Santa Maria
SID	Once a Day
H&E	Hematoxylin and Eosin
USA	United States of America
